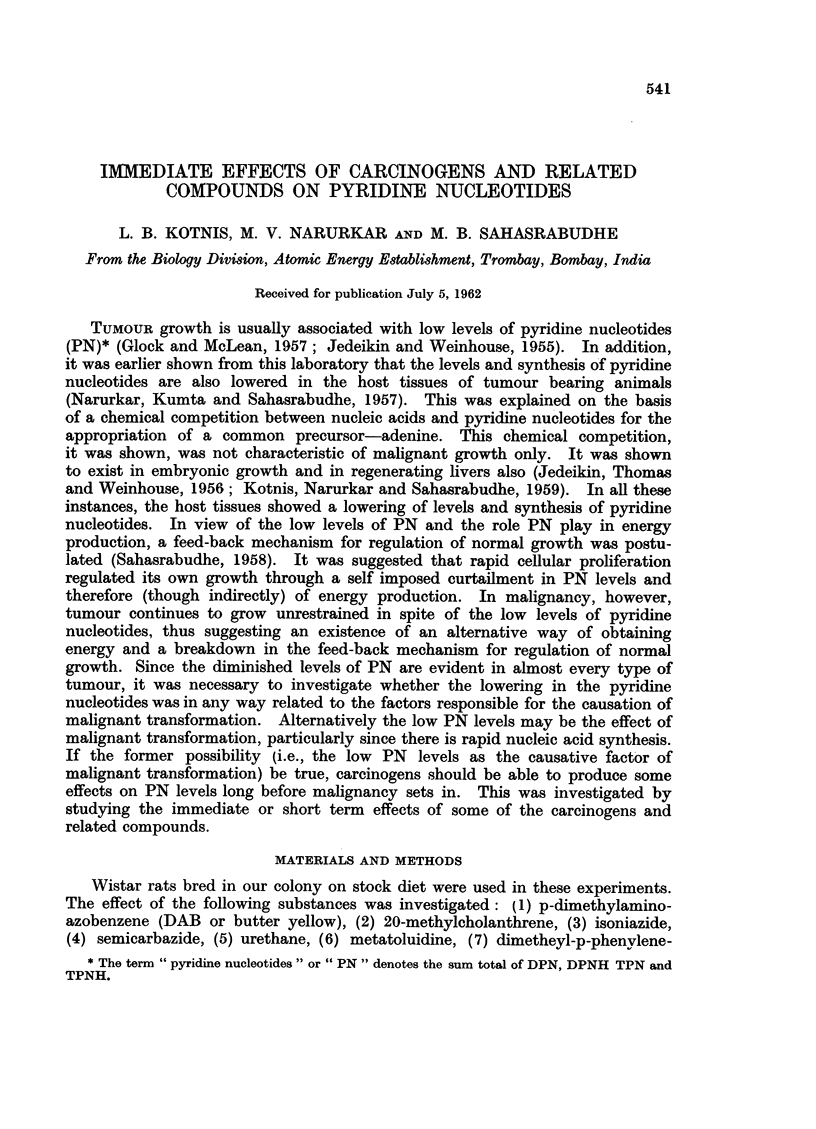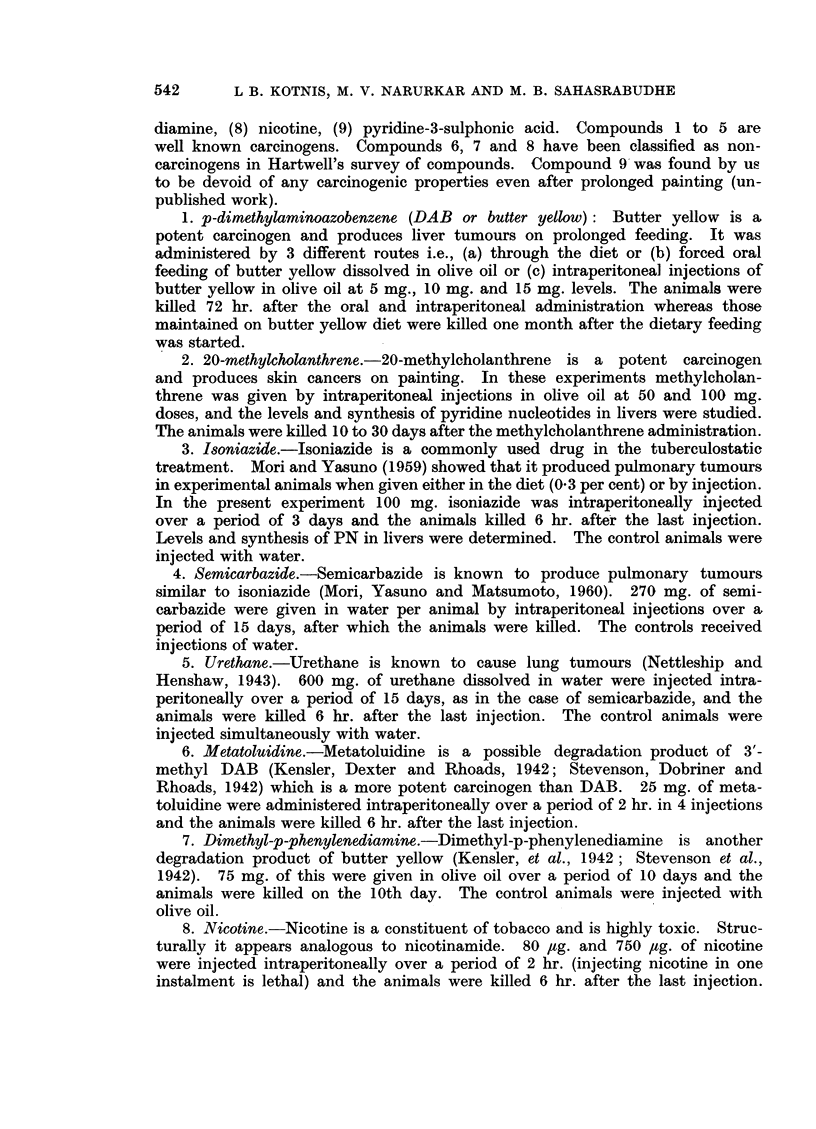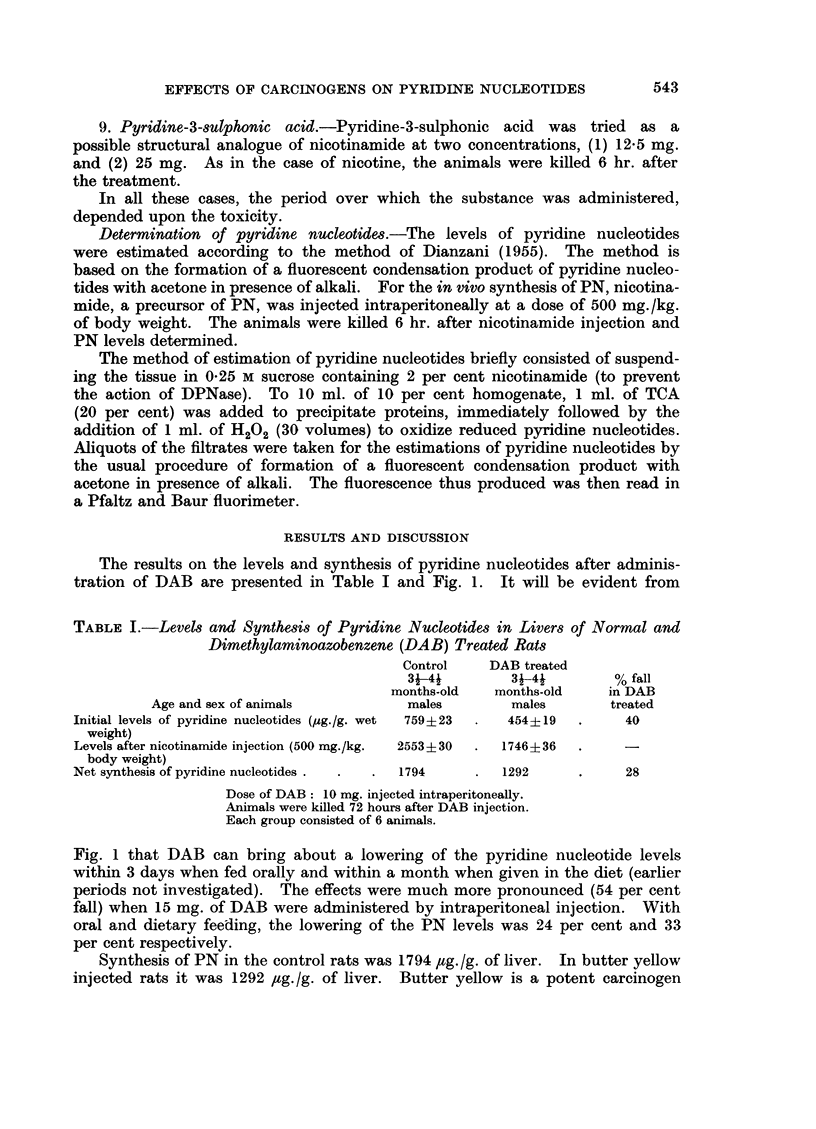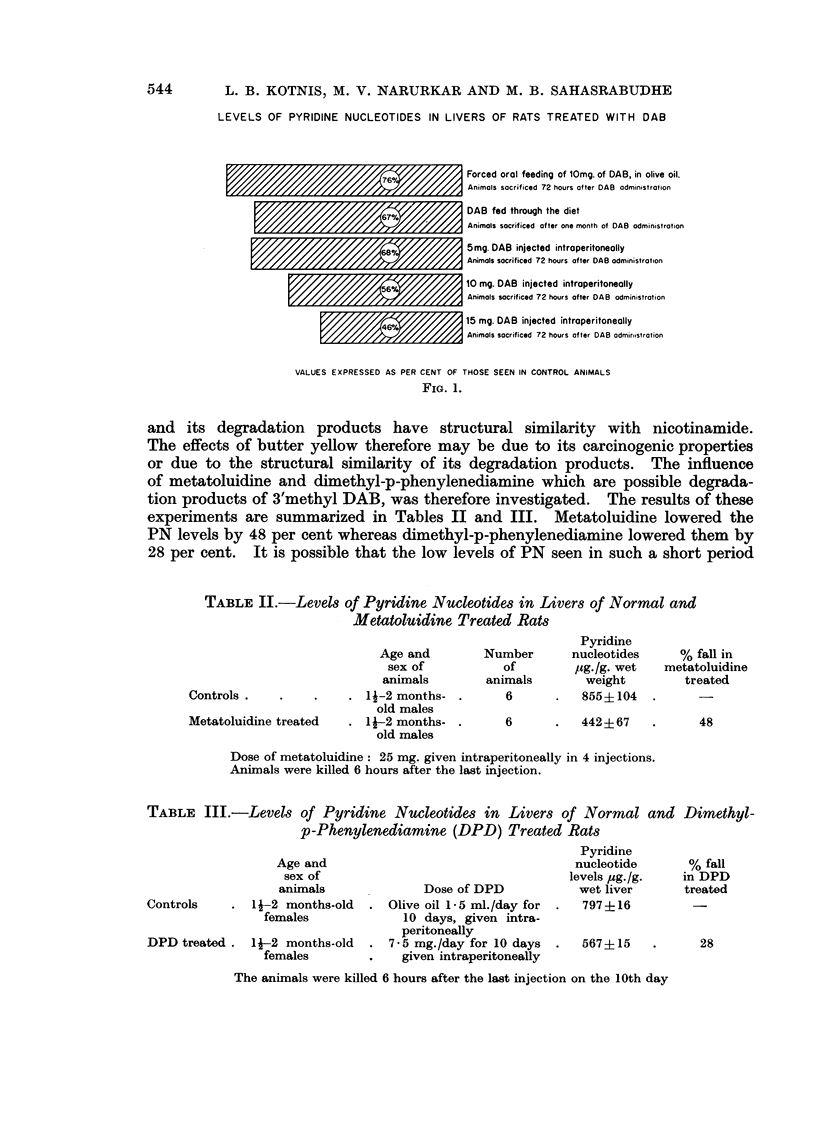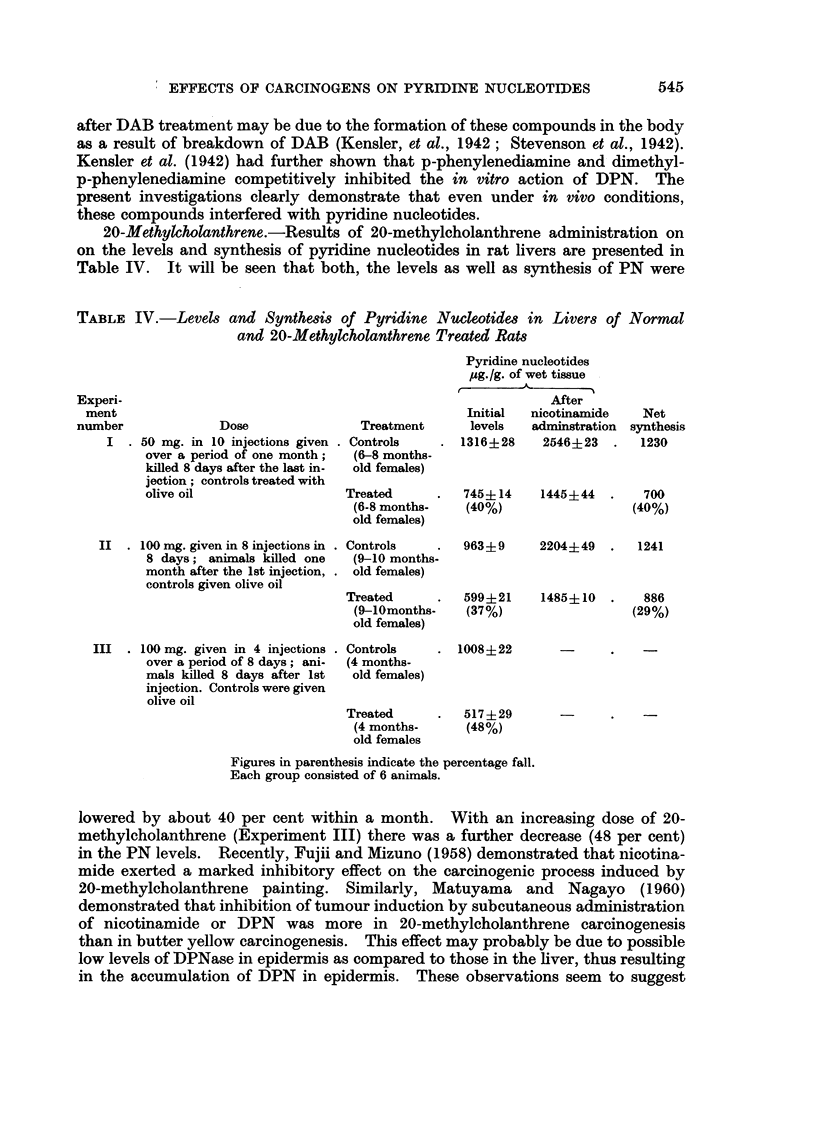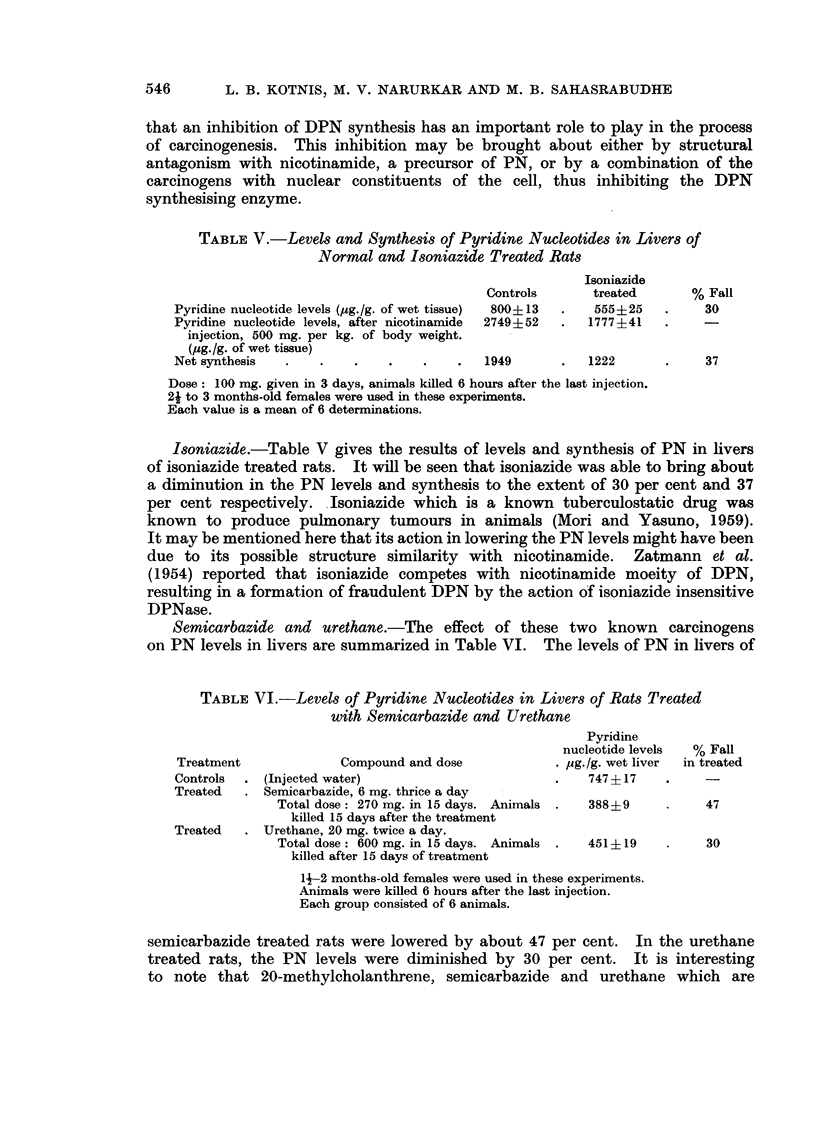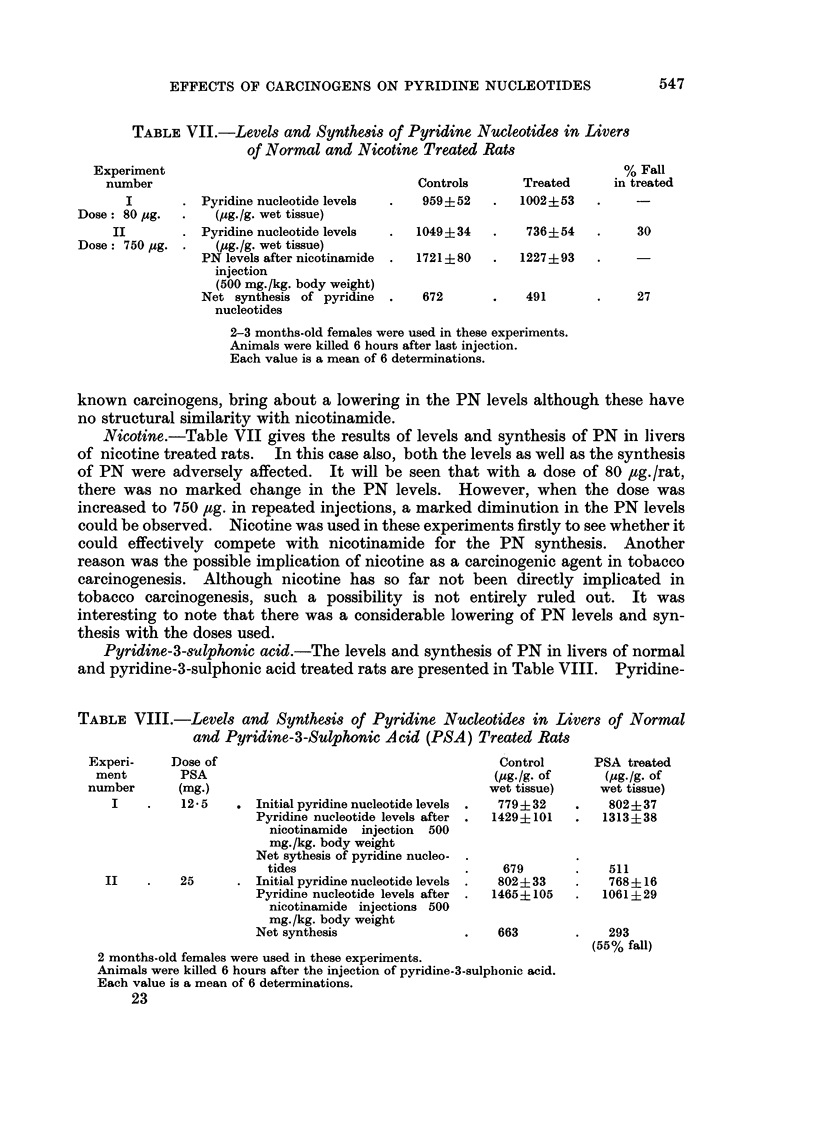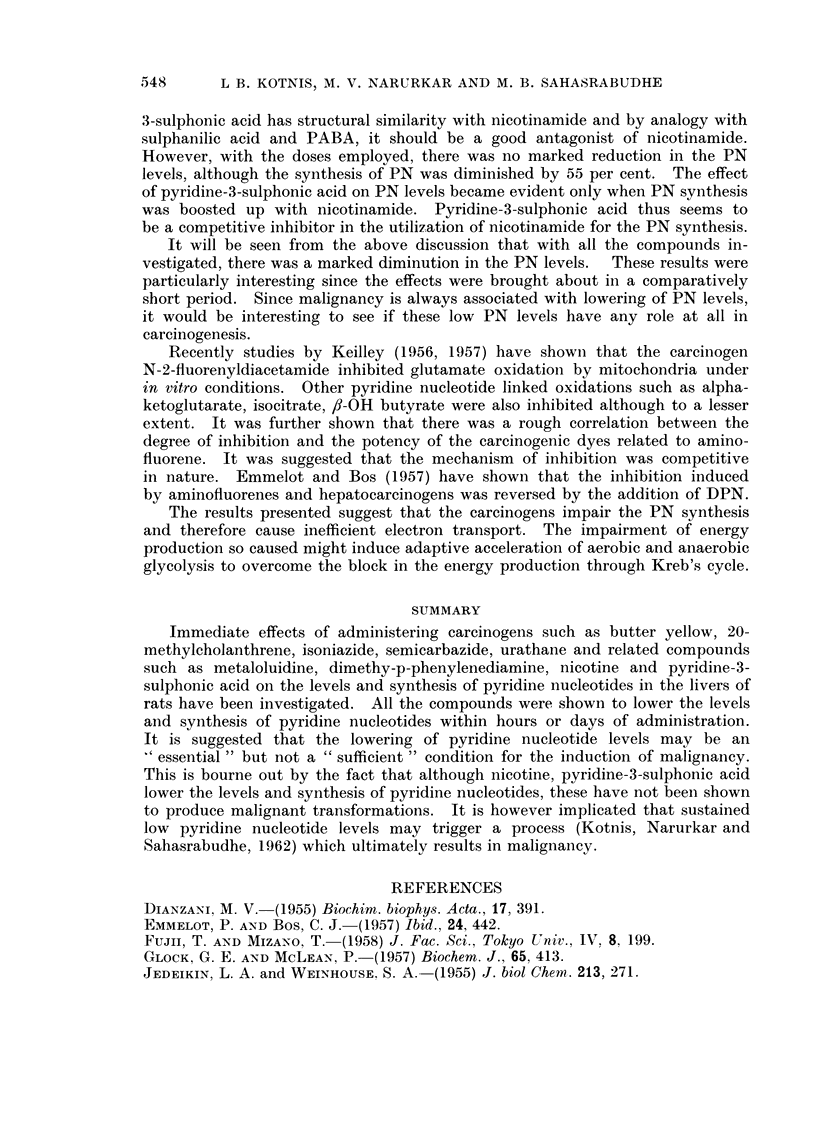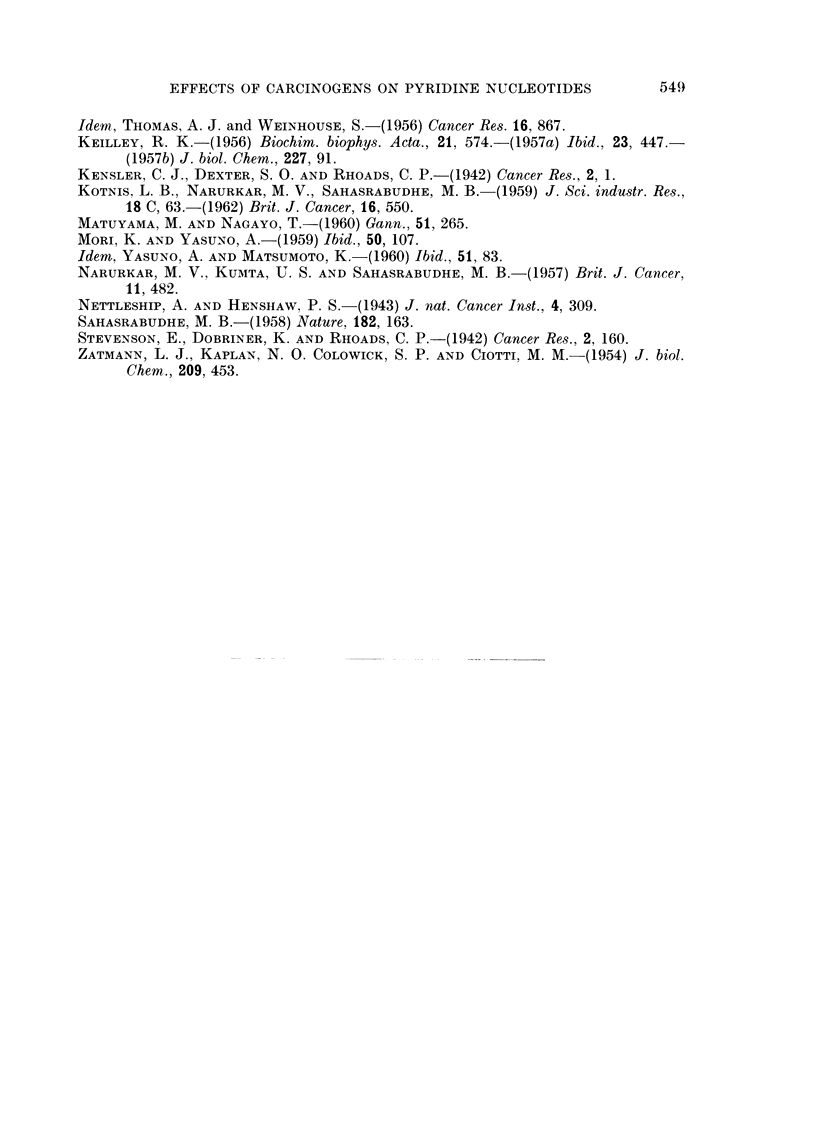# Immediate Effects of Carcinogens and Related Compounds on Pyridine Nucleotides

**DOI:** 10.1038/bjc.1962.62

**Published:** 1962-09

**Authors:** L. B. Kotnis, M. V. Narurkar, M. B. Sahasrabudhe


### IMMEDIATE EFFECTS OF CARCINOGENS AND RELATED COMPOUNDS ON PYRIDINE NUCLEOTIDES

L. B. KOTNIS, M. V. NARURKAR AND M. B. SAHASRABUDHE

*From the Biology Division, Atomic Energy Establishment, Trombay, Bombay, India*

Received for publication July 5, 1962

TUMOUR growth is usually associated with low levels of pyridine nucleotides (PN)* (Glock and McLean, 1957 ; Jedeikin and Weinhouse, 1955). In addition, it was earlier shown from this laboratory that the levels and synthesis of pyridine nucleotides are also lowered in the host tissues of tumour bearing animals (Narurkar, Kumta and Sahasrabudhe, 1957). This was explained on the basis of a chemical competition between nucleic acids and pyridine nucleotides for the appropriation of a common precursor—adenine. This chemical competition, it was shown, was not characteristic of malignant growth only. It was shown to exist in embryonic growth and in regenerating livers also (Jedeikin, Thomas and Weinhouse, 1956 ; Kotnis, Narurkar and Sahasrabudhe, 1959). In all these instances, the host tissues showed a lowering of levels and synthesis of pyridine nucleotides. In view of the low levels of PN and the role PN play in energy production, a feed-back mechanism for regulation of normal growth was postulated (Sahasrabudhe, 1958). It was suggested that rapid cellular proliferation regulated its own growth through a self imposed curtailment in PN levels and therefore (though indirectly) of energy production. In malignancy, however, tumour continues to grow unrestrained in spite of the low levels of pyridine nucleotides, thus suggesting an existence of an alternative way of obtaining energy and a breakdown in the feed-back mechanism for regulation of normal growth. Since the diminished levels of PN are evident in almost every type of tumour, it was necessary to investigate whether the lowering in the pyridine nucleotides was in any way related to the factors responsible for the causation of malignant transformation. Alternatively the low PN levels may be the effect of malignant transformation, particularly since there is rapid nucleic acid synthesis. If the former possibility (i.e., the low PN levels as the causative factor of malignant transformation) be true, carcinogens should be able to produce some effects on PN levels long before malignancy sets in. This was investigated by studying the immediate or short term effects of some of the carcinogens and related compounds.

### MATERIALS AND METHODS

Wistar rats bred in our colony on stock diet were used in these experiments. The effect of the following substances was investigated : (1) p-dimethylamino-azobenzene (DAB or butter yellow), (2) 20-methylcholanthrene, (3) isoniazide, (4) semicarbazide, (5) urethane, (6) metatoluidine, (7) dimetheyl-p-phenylene-

* The term " pyridine nucleotides " or " PN " denotes the sum total of DPN, DPNH TPN and TPNH.

diamine, (8) nicotine, (9) pyridine-3-sulphonic acid. Compounds 1 to 5 are well known carcinogens. Compounds 6, 7 and 8 have been classified as non-carcinogens in Hartwell's survey of compounds. Compound 9 was found by us to be devoid of any carcinogenic properties even after prolonged painting (unpublished work).

1. *p-dimethylaminoazobenzene* (*DAB or butter yellow*): Butter yellow is a potent carcinogen and produces liver tumours on prolonged feeding. It was administered by 3 different routes i.e., (a) through the diet or (b) forced oral feeding of butter yellow dissolved in olive oil or (c) intraperitoneal injections of butter yellow in olive oil at 5 mg., 10 mg. and 15 mg. levels. The animals were killed 72 hr. after the oral and intraperitoneal administration whereas those maintained on butter yellow diet were killed one month after the dietary feeding was started.

2. 20-*methylcholanthrene*.—20-methylcholanthrene is a potent carcinogen and produces skin cancers on painting. In these experiments methylcholanthrene was given by intraperitoneal injections in olive oil at 50 and 100 mg. doses, and the levels and synthesis of pyridine nucleotides in livers were studied. The animals were killed 10 to 30 days after the methylcholanthrene administration.

3. *Isoniazide*.—Isoniazide is a commonly used drug in the tuberculostatic treatment. Mori and Yasuno (1959) showed that it produced pulmonary tumours in experimental animals when given either in the diet (0·3 per cent) or by injection. In the present experiment 100 mg. isoniazide was intraperitoneally injected over a period of 3 days and the animals killed 6 hr. after the last injection. Levels and synthesis of PN in livers were determined. The control animals were injected with water.

4. *Semicarbazide*.—Semicarbazide is known to produce pulmonary tumours similar to isoniazide (Mori, Yasuno and Matsumoto, 1960). 270 mg. of semicarbazide were given in water per animal by intraperitoneal injections over a period of 15 days, after which the animals were killed. The controls received injections of water.

5. *Urethane*.—Urethane is known to cause lung tumours (Nettleship and Henshaw, 1943). 600 mg. of urethane dissolved in water were injected intraperitoneally over a period of 15 days, as in the case of semicarbazide, and the animals were killed 6 hr. after the last injection. The control animals were injected simultaneously with water.

6. *Metatoluidine*.—Metatoluidine is a possible degradation product of 3′-methyl DAB (Kensler, Dexter and Rhoads, 1942; Stevenson, Dobriner and Rhoads, 1942) which is a more potent carcinogen than DAB. 25 mg. of metatoluidine were administered intraperitoneally over a period of 2 hr. in 4 injections and the animals were killed 6 hr. after the last injection.

7. *Dimethyl-p-phenylenediamine*.—Dimethyl-p-phenylenediamine is another degradation product of butter yellow (Kensler, *et al.*, 1942; Stevenson *et al.*, 1942). 75 mg. of this were given in olive oil over a period of 10 days and the animals were killed on the 10th day. The control animals were injected with olive oil.

8. *Nicotine*.—Nicotine is a constituent of tobacco and is highly toxic. Structurally it appears analogous to nicotinamide. 80 $\mu$g. and 750 $\mu$g. of nicotine were injected intraperitoneally over a period of 2 hr. (injecting nicotine in one instalment is lethal) and the animals were killed 6 hr. after the last injection.

9. *Pyridine-3-sulphonic acid.*—Pyridine-3-sulphonic acid was tried as a possible structural analogue of nicotinamide at two concentrations, (1) 12·5 mg. and (2) 25 mg. As in the case of nicotine, the animals were killed 6 hr. after the treatment.

In all these cases, the period over which the substance was administered, depended upon the toxicity.

*Determination of pyridine nucleotides.*—The levels of pyridine nucleotides were estimated according to the method of Dianzani (1955). The method is based on the formation of a fluorescent condensation product of pyridine nucleotides with acetone in presence of alkali. For the *in vivo* synthesis of PN, nicotinamide, a precursor of PN, was injected intraperitoneally at a dose of 500 mg./kg. of body weight. The animals were killed 6 hr. after nicotinamide injection and PN levels determined.

The method of estimation of pyridine nucleotides briefly consisted of suspending the tissue in 0·25 M sucrose containing 2 per cent nicotinamide (to prevent the action of DPNase). To 10 ml. of 10 per cent homogenate, 1 ml. of TCA (20 per cent) was added to precipitate proteins, immediately followed by the addition of 1 ml. of $H_2O_2$ (30 volumes) to oxidize reduced pyridine nucleotides. Aliquots of the filtrates were taken for the estimations of pyridine nucleotides by the usual procedure of formation of a fluorescent condensation product with acetone in presence of alkali. The fluorescence thus produced was then read in a Pfaltz and Baur fluorimeter.

### RESULTS AND DISCUSSION

The results on the levels and synthesis of pyridine nucleotides after administration of DAB are presented in Table I and Fig. 1. It will be evident from

TABLE I.—*Levels and Synthesis of Pyridine Nucleotides in Livers of Normal and Dimethylaminoazobenzene (DAB) Treated Rats*

| Age and sex of animals | Control 3½–4½ months-old males | DAB treated 3½–4½ months-old males | % fall in DAB treated |
|---|---|---|---|
| Initial levels of pyridine nucleotides (μg./g. wet weight) | 759±23 | 454±19 | 40 |
| Levels after nicotinamide injection (500 mg./kg. body weight) | 2553±30 | 1746±36 | — |
| Net synthesis of pyridine nucleotides | 1794 | 1292 | 28 |

Dose of DAB : 10 mg. injected intraperitoneally.
Animals were killed 72 hours after DAB injection.
Each group consisted of 6 animals.

Fig. 1 that DAB can bring about a lowering of the pyridine nucleotide levels within 3 days when fed orally and within a month when given in the diet (earlier periods not investigated). The effects were much more pronounced (54 per cent fall) when 15 mg. of DAB were administered by intraperitoneal injection. With oral and dietary feeding, the lowering of the PN levels was 24 per cent and 33 per cent respectively.

Synthesis of PN in the control rats was 1794 μg./g. of liver. In butter yellow injected rats it was 1292 μg./g. of liver. Butter yellow is a potent carcinogen

LEVELS OF PYRIDINE NUCLEOTIDES IN LIVERS OF RATS TREATED WITH DAB

VALUES EXPRESSED AS PER CENT OF THOSE SEEN IN CONTROL ANIMALS

FIG. 1.

and its degradation products have structural similarity with nicotinamide. The effects of butter yellow therefore may be due to its carcinogenic properties or due to the structural similarity of its degradation products. The influence of metatoluidine and dimethyl-p-phenylenediamine which are possible degradation products of 3′methyl DAB, was therefore investigated. The results of these experiments are summarized in Tables II and III. Metatoluidine lowered the PN levels by 48 per cent whereas dimethyl-p-phenylenediamine lowered them by 28 per cent. It is possible that the low levels of PN seen in such a short period

TABLE II.—*Levels of Pyridine Nucleotides in Livers of Normal and Metatoluidine Treated Rats*

| | Age and sex of animals | Number of animals | Pyridine nucleotides μg./g. wet weight | % fall in metatoluidine treated |
|---|---|---|---|---|
| Controls | 1½–2 months-old males | 6 | 855±104 | — |
| Metatoluidine treated | 1½–2 months-old males | 6 | 442±67 | 48 |

Dose of metatoluidine : 25 mg. given intraperitoneally in 4 injections. Animals were killed 6 hours after the last injection.

TABLE III.—*Levels of Pyridine Nucleotides in Livers of Normal and Dimethyl-p-Phenylenediamine (DPD) Treated Rats*

| | Age and sex of animals | Dose of DPD | Pyridine nucleotide levels μg./g. wet liver | % fall in DPD treated |
|---|---|---|---|---|
| Controls | 1½–2 months-old females | Olive oil 1·5 ml./day for 10 days, given intraperitoneally | 797±16 | — |
| DPD treated | 1½–2 months-old females | 7·5 mg./day for 10 days given intraperitoneally | 567±15 | 28 |

The animals were killed 6 hours after the last injection on the 10th day after DAB treatment may be due to the formation of these compounds in the body as a result of breakdown of DAB (Kensler, *et al.*, 1942 ; Stevenson *et al.*, 1942). Kensler *et al.* (1942) had further shown that p-phenylenediamine and dimethyl-p-phenylenediamine competitively inhibited the *in vitro* action of DPN. The present investigations clearly demonstrate that even under *in vivo* conditions, these compounds interfered with pyridine nucleotides.

20-*Methylcholanthrene*.—Results of 20-methylcholanthrene administration on on the levels and synthesis of pyridine nucleotides in rat livers are presented in Table IV. It will be seen that both, the levels as well as synthesis of PN were

TABLE IV.—*Levels and Synthesis of Pyridine Nucleotides in Livers of Normal and 20-Methylcholanthrene Treated Rats*

| Experiment number | Dose | Treatment | Pyridine nucleotides µg./g. of wet tissue: Initial levels | Pyridine nucleotides µg./g. of wet tissue: After nicotinamide adminstration | Net synthesis |
|---|---|---|---|---|---|
| I | 50 mg. in 10 injections given over a period of one month ; killed 8 days after the last injection ; controls treated with olive oil | Controls (6–8 months-old females) | 1316±28 | 2546±23 | 1230 |
| | | Treated (6-8 months-old females) | 745±14 (40%) | 1445±44 | 700 (40%) |
| II | 100 mg. given in 8 injections in 8 days ; animals killed one month after the 1st injection, controls given olive oil | Controls (9–10 months-old females) | 963±9 | 2204±49 | 1241 |
| | | Treated (9–10months-old females) | 599±21 (37%) | 1485±10 | 886 (29%) |
| III | 100 mg. given in 4 injections over a period of 8 days ; animals killed 8 days after 1st injection. Controls were given olive oil | Controls (4 months-old females) | 1008±22 | — | — |
| | | Treated (4 months-old females | 517±29 (48%) | — | — |

Figures in parenthesis indicate the percentage fall.
Each group consisted of 6 animals.

lowered by about 40 per cent within a month. With an increasing dose of 20-methylcholanthrene (Experiment III) there was a further decrease (48 per cent) in the PN levels. Recently, Fujii and Mizuno (1958) demonstrated that nicotinamide exerted a marked inhibitory effect on the carcinogenic process induced by 20-methylcholanthrene painting. Similarly, Matuyama and Nagayo (1960) demonstrated that inhibition of tumour induction by subcutaneous administration of nicotinamide or DPN was more in 20-methylcholanthrene carcinogenesis than in butter yellow carcinogenesis. This effect may probably be due to possible low levels of DPNase in epidermis as compared to those in the liver, thus resulting in the accumulation of DPN in epidermis. These observations seem to suggest that an inhibition of DPN synthesis has an important role to play in the process of carcinogenesis. This inhibition may be brought about either by structural antagonism with nicotinamide, a precursor of PN, or by a combination of the carcinogens with nuclear constituents of the cell, thus inhibiting the DPN synthesising enzyme.

TABLE V.—*Levels and Synthesis of Pyridine Nucleotides in Livers of Normal and Isoniazide Treated Rats*

| | Controls | Isoniazide treated | % Fall |
|---|---|---|---|
| Pyridine nucleotide levels (μg./g. of wet tissue) | 800±13 | 555±25 | 30 |
| Pyridine nucleotide levels, after nicotinamide injection, 500 mg. per kg. of body weight. (μg./g. of wet tissue) | 2749±52 | 1777±41 | — |
| Net synthesis | 1949 | 1222 | 37 |

Dose : 100 mg. given in 3 days, animals killed 6 hours after the last injection.
2½ to 3 months-old females were used in these experiments.
Each value is a mean of 6 determinations.

*Isoniazide.*—Table V gives the results of levels and synthesis of PN in livers of isoniazide treated rats. It will be seen that isoniazide was able to bring about a diminution in the PN levels and synthesis to the extent of 30 per cent and 37 per cent respectively. Isoniazide which is a known tuberculostatic drug was known to produce pulmonary tumours in animals (Mori and Yasuno, 1959). It may be mentioned here that its action in lowering the PN levels might have been due to its possible structure similarity with nicotinamide. Zatmann *et al.* (1954) reported that isoniazide competes with nicotinamide moeity of DPN, resulting in a formation of fraudulent DPN by the action of isoniazide insensitive DPNase.

*Semicarbazide and urethane.*—The effect of these two known carcinogens on PN levels in livers are summarized in Table VI. The levels of PN in livers of

TABLE VI.—*Levels of Pyridine Nucleotides in Livers of Rats Treated with Semicarbazide and Urethane*

| Treatment | Compound and dose | Pyridine nucleotide levels μg./g. wet liver | % Fall in treated |
|---|---|---|---|
| Controls | (Injected water) | 747±17 | — |
| Treated | Semicarbazide, 6 mg. thrice a day. Total dose : 270 mg. in 15 days. Animals killed 15 days after the treatment | 388±9 | 47 |
| Treated | Urethane, 20 mg. twice a day. Total dose : 600 mg. in 15 days. Animals killed after 15 days of treatment | 451±19 | 30 |

1½–2 months-old females were used in these experiments.
Animals were killed 6 hours after the last injection.
Each group consisted of 6 animals.

semicarbazide treated rats were lowered by about 47 per cent. In the urethane treated rats, the PN levels were diminished by 30 per cent. It is interesting to note that 20-methylcholanthrene, semicarbazide and urethane which are

TABLE VII.—*Levels and Synthesis of Pyridine Nucleotides in Livers of Normal and Nicotine Treated Rats*

| Experiment number | | Controls | Treated | % Fall in treated |
|---|---|---|---|---|
| I Dose: 80 $\mu$g. | Pyridine nucleotide levels ($\mu$g./g. wet tissue) | 959±52 | 1002±53 | — |
| II Dose: 750 $\mu$g. | Pyridine nucleotide levels ($\mu$g./g. wet tissue) | 1049±34 | 736±54 | 30 |
| | PN levels after nicotinamide injection (500 mg./kg. body weight) | 1721±80 | 1227±93 | — |
| | Net synthesis of pyridine nucleotides | 672 | 491 | 27 |

2–3 months-old females were used in these experiments.
Animals were killed 6 hours after last injection.
Each value is a mean of 6 determinations.

known carcinogens, bring about a lowering in the PN levels although these have no structural similarity with nicotinamide.

*Nicotine.*—Table VII gives the results of levels and synthesis of PN in livers of nicotine treated rats. In this case also, both the levels as well as the synthesis of PN were adversely affected. It will be seen that with a dose of 80 $\mu$g./rat, there was no marked change in the PN levels. However, when the dose was increased to 750 $\mu$g. in repeated injections, a marked diminution in the PN levels could be observed. Nicotine was used in these experiments firstly to see whether it could effectively compete with nicotinamide for the PN synthesis. Another reason was the possible implication of nicotine as a carcinogenic agent in tobacco carcinogenesis. Although nicotine has so far not been directly implicated in tobacco carcinogenesis, such a possibility is not entirely ruled out. It was interesting to note that there was a considerable lowering of PN levels and synthesis with the doses used.

*Pyridine-3-sulphonic acid.*—The levels and synthesis of PN in livers of normal and pyridine-3-sulphonic acid treated rats are presented in Table VIII. Pyridine-

TABLE VIII.—*Levels and Synthesis of Pyridine Nucleotides in Livers of Normal and Pyridine-3-Sulphonic Acid (PSA) Treated Rats*

| Experiment number | Dose of PSA (mg.) | | Control ($\mu$g./g. of wet tissue) | PSA treated ($\mu$g./g. of wet tissue) |
|---|---|---|---|---|
| I | 12·5 | Initial pyridine nucleotide levels | 779±32 | 802±37 |
| | | Pyridine nucleotide levels after nicotinamide injection 500 mg./kg. body weight | 1429±101 | 1313±38 |
| | | Net sythesis of pyridine nucleotides | 679 | 511 |
| II | 25 | Initial pyridine nucleotide levels | 802±33 | 768±16 |
| | | Pyridine nucleotide levels after nicotinamide injections 500 mg./kg. body weight | 1465±105 | 1061±29 |
| | | Net synthesis | 663 | 293 (55% fall) |

2 months-old females were used in these experiments.
Animals were killed 6 hours after the injection of pyridine-3-sulphonic acid.
Each value is a mean of 6 determinations.

3-sulphonic acid has structural similarity with nicotinamide and by analogy with sulphanilic acid and PABA, it should be a good antagonist of nicotinamide. However, with the doses employed, there was no marked reduction in the PN levels, although the synthesis of PN was diminished by 55 per cent. The effect of pyridine-3-sulphonic acid on PN levels became evident only when PN synthesis was boosted up with nicotinamide. Pyridine-3-sulphonic acid thus seems to be a competitive inhibitor in the utilization of nicotinamide for the PN synthesis.

It will be seen from the above discussion that with all the compounds investigated, there was a marked diminution in the PN levels. These results were particularly interesting since the effects were brought about in a comparatively short period. Since malignancy is always associated with lowering of PN levels, it would be interesting to see if these low PN levels have any role at all in carcinogenesis.

Recently studies by Keilley (1956, 1957) have shown that the carcinogen N-2-fluorenyldiacetamide inhibited glutamate oxidation by mitochondria under *in vitro* conditions. Other pyridine nucleotide linked oxidations such as alpha-ketoglutarate, isocitrate, $\beta$-OH butyrate were also inhibited although to a lesser extent. It was further shown that there was a rough correlation between the degree of inhibition and the potency of the carcinogenic dyes related to aminofluorene. It was suggested that the mechanism of inhibition was competitive in nature. Emmelot and Bos (1957) have shown that the inhibition induced by aminofluorenes and hepatocarcinogens was reversed by the addition of DPN.

The results presented suggest that the carcinogens impair the PN synthesis and therefore cause inefficient electron transport. The impairment of energy production so caused might induce adaptive acceleration of aerobic and anaerobic glycolysis to overcome the block in the energy production through Kreb's cycle.

### SUMMARY

Immediate effects of administering carcinogens such as butter yellow, 20-methylcholanthrene, isoniazide, semicarbazide, urathane and related compounds such as metaloluidine, dimethy-p-phenylenediamine, nicotine and pyridine-3-sulphonic acid on the levels and synthesis of pyridine nucleotides in the livers of rats have been investigated. All the compounds were shown to lower the levels and synthesis of pyridine nucleotides within hours or days of administration. It is suggested that the lowering of pyridine nucleotide levels may be an " essential " but not a " sufficient " condition for the induction of malignancy. This is bourne out by the fact that although nicotine, pyridine-3-sulphonic acid lower the levels and synthesis of pyridine nucleotides, these have not been shown to produce malignant transformations. It is however implicated that sustained low pyridine nucleotide levels may trigger a process (Kotnis, Narurkar and Sahasrabudhe, 1962) which ultimately results in malignancy.

### REFERENCES

DIANZANI, M. V.—(1955) *Biochim. biophys. Acta.*, **17**, 391.
EMMELOT, P. AND BOS, C. J.—(1957) *Ibid.*, **24**, 442.
FUJII, T. AND MIZANO, T.—(1958) *J. Fac. Sci., Tokyo Univ.*, IV, **8**, 199.
GLOCK, G. E. AND MCLEAN, P.—(1957) *Biochem. J.*, **65**, 413.
JEDEIKIN, L. A. and WEINHOUSE, S. A.—(1955) *J. biol Chem.* **213**, 271.

*Idem*, Thomas, A. J. and Weinhouse, S.—(1956) *Cancer Res.* **16**, 867.
Keilley, R. K.—(1956) *Biochim. biophys. Acta.*, **21**, 574.—(1957*a*) *Ibid.*, **23**, 447.—(1957*b*) *J. biol. Chem.*, **227**, 91.
Kensler, C. J., Dexter, S. O. and Rhoads, C. P.—(1942) *Cancer Res.*, **2**, 1.
Kotnis, L. B., Narurkar, M. V., Sahasrabudhe, M. B.—(1959) *J. Sci. industr. Res.*, **18** C, 63.—(1962) *Brit. J. Cancer*, **16**, 550.
Matuyama, M. and Nagayo, T.—(1960) *Gann.*, **51**, 265.
Mori, K. and Yasuno, A.—(1959) *Ibid.*, **50**, 107.
*Idem*, Yasuno, A. and Matsumoto, K.—(1960) *Ibid.*, **51**, 83.
Narurkar, M. V., Kumta, U. S. and Sahasrabudhe, M. B.—(1957) *Brit. J. Cancer*, **11**, 482.
Nettleship, A. and Henshaw, P. S.—(1943) *J. nat. Cancer Inst.*, **4**, 309.
Sahasrabudhe, M. B.—(1958) *Nature*, **182**, 163.
Stevenson, E., Dobriner, K. and Rhoads, C. P.—(1942) *Cancer Res.*, **2**, 160.
Zatmann, L. J., Kaplan, N. O. Colowick, S. P. and Ciotti, M. M.—(1954) *J. biol. Chem.*, **209**, 453.